# Comment on McCarthy, C.; et al. Developing Picornaviruses for Cancer Therapy. *Cancers* 2019, *11*, 685

**DOI:** 10.3390/cancers12071775

**Published:** 2020-07-03

**Authors:** Pēteris Alberts

**Affiliations:** Rigvir, Atlasa iela 7C, LV-1026 Riga, Latvia; peteris.alberts@rigvir.com

I would like to make the following corrections to the publication by McCarthy et al. [1], for the review to give correct information on the oncolytic ECHO-7 virus Rigvir.

1. On page 14, chapter 4, paragraph 2, line 6, the authors wrote: “The United States, much of the European Union and Japan found a lack of experimental evidence to approve Rigvir in cancer treatment [99].”

However, Rigvir has not been submitted for registration to the agencies of the mentioned countries. The Rigvir drug development plans are known to the European Medicines Agency (EMA).

Therefore, the sentence cited above should be deleted.

2. On page 14, chapter 4, paragraph 4, line 1, the authors wrote: “Clinical trials are referred to in the literature to have occurred between 1965 and 1991, but the results from these clinical trials are not readily available [96].”

However, “The major part of the Rigvir pre-registration clinical studies was performed during the period from 1968 to 1991.” [2].

Therefore, the sentence should read: “Clinical trials are referred to in the literature to have occurred between 1968 and 1991, but the results from these clinical trials are not readily available [96].”

3. On page 14, paragraph 4, line 4, the authors wrote: “It is believed that in Latvia, 75% of melanoma cases are treated with Rigvir [98]. Other Eastern European countries such as Georgia, Armenia and Uzbekistan have also approved Rigvir [96].” See also page 21 and Table 3.

However, for some time now, the United Nations have characterized Latvia, as well as Estonia, Lithuania, the Scandinavian countries and the UK as being part of Northern Europe [3].

Therefore, the sentence should read: “It is believed that in Latvia, 75% of melanoma cases are treated with Rigvir [98]. Eastern European countries such as Georgia, Armenia and Uzbekistan have also approved Rigvir [96].”

4. On page 15, line 1, the authors wrote: “One such retrospective study showed a statistically significant increase in 3-year survival of melanoma patients when treated with Rigvir post-surgery than surgery alone or with other immunomodulators [97].”

However, in the cited reference, 3-year survival data were not determined. In contrast, it was stated that “These results indicate that the patients treated with Rigvir had a 4.39–6.57-fold lower mortality than those under observation” [4]. Furthermore, the effect of other immunomodulators was not determined.

Therefore, the sentence should read: “One such retrospective study showed a statistically significant 4.39–6.57-fold decrease in mortality of melanoma patients when treated with Rigvir post-surgery rather than surgery alone [97].”

5. On page 15, line 3, the authors wrote: “For best efficacy, Rigvir is injected intratumourally rather than intramuscularly, increasing 5-year survival between 29.9% and 19.5% [97].”

However, the registered mode of administration of Rigvir is intramuscular [2,3,4,5,6,7,8].

Therefore, the sentence cited above should be deleted.

6. On page 15, line 4, the authors wrote: “Regional inoculation of Rigvir increases the total proportion of active (CD38+) and cytotoxic (CD8+) T-cells [100].”

However, the effect of Rigvir on lymphocyte subpopulations is shown after in vitro treatment with Rigvir (Table 3 of [5]). 

Therefore, the sentence should read: “Rigvir increases the total proportion of active (CD38+) and cytotoxic (CD8+) T-cells [100].”

7. On page 15, line 5, the authors wrote: “Another retrospective trial focused on melanoma patients Stage IB through to Stage IIC, with 52 patients enrolled for Rigvir therapy and 27 electing to go without [97].”

However, this is the same study that has already been discussed and referred to once on page 14 and twice on page 15.

Therefore, the sentence should read: “The retrospective study focused on melanoma patients Stage IB through to Stage IIC, with 52 patients enrolled for Rigvir therapy and 27 electing to go without therapy [97].”

8. On page 15, paragraph 2, line 5, the authors wrote: “The small-cell lung cancer patient was treated with lariphan and Rigvir but at a constant infrequent schedule for six years.”

However, the patient was not treated with lariphan for six years, only weekly for a month [6].

Therefore, the sentence should read: “The small-cell lung cancer patient was treated with lariphan weekly for a month and then with Rigvir, but at a constant infrequent schedule for six years.”

9. On page 15, paragraph 2, line 7, the authors wrote: “In the case of the stage IV histiocytic sarcoma patient, Rigvir was administered as part of a therapeutic cocktail including multisite radiation therapy, doxorubicin, cyclophosphamide and helixor P.”

However, the treatment of this patient started with Rigvir for 2 years. Only then did he receive radiation therapy and chemotherapy (see Figure 9 of [6]).

Therefore, the sentence should read: “In the case of the stage IV histiocytic sarcoma patient, Rigvir was administered for about 2 years before start of classical radiation therapy and chemotherapy.”

10. On page 15, paragraph 3, line 2, the authors wrote: “One patient with a basal cell carcinoma, a diagnosis with a median expected survival of five months, was treated with Rigvir post-surgery [101].”

However, there is no histological record available confirming the basal cell carcinoma diagnosis. In contrast, immunohistochemistry showed that the tumor cells were weakly positive for HMB-45 and Melan A, strongly positive for MART-1, S-100 and vimentin, and that the Ki-67 index was 35% [7].

Therefore, the sentence should read: “One patient with melanoma unknown primary brain metastasis, a diagnosis with a median expected survival of five months, was treated with Rigvir post-surgery [101].”

11. On page 15, paragraph 3, line 7, the authors wrote: “[102]. The patient was first treated with 11 injections of Rigvir in combination with eight infusions with folinic acid-fluorouracil-oxiplatin (FOLFOX-4) and four infusions of bevacizumab.”

However, the patient was first treated with FOLFOX-4 and bevacizumab, and in addition with Rigvir [8].

Therefore, the sentence should read: “[102]. The patient was treated with a combination of eight infusions of folinic acid-fluorouracil-oxiplatin (FOLFOX-4), four infusions of bevacizumab, and with 11 injections of Rigvir over 13 months intermittently between the chemotherapy cycles.”
